# Preoperative Physical Activity Level Has No Relationship to the Degree of Recovery One Year after Primary Total Hip or Knee Arthroplasty: A Cohort Study

**DOI:** 10.1371/journal.pone.0115559

**Published:** 2014-12-23

**Authors:** Sietske Poortinga, Inge van den Akker-Scheek, Sjoerd K. Bulstra, Roy E. Stewart, Martin Stevens

**Affiliations:** 1 Department of Orthopaedics, University of Groningen, University Medical Center Groningen, Groningen, The Netherlands; 2 Department of Health Sciences, Community and Occupational Sciences, University of Groningen, University Medical Center Groningen, Groningen, The Netherlands; The University of Queensland, Australia

## Abstract

**Background:**

When it comes to prevalidation, it is assumed that a higher preoperative level of physical activity leads to better postoperative recovery. However current literature is inconclusive about the effect of prevalidation on functional recovery of patients with primary osteoarthritis (OA) who underwent a THA or TKA. Therefore the aim of this study is to analyse one of the major assumptions underlying the potential effectiveness of prevalidation namely the relationship between preoperative physical activity level and postoperative recovery one year after THA or TKA in a group of 658 OA patients.

**Methods and Results:**

From 2006 to 2012, 1061 patients underwent a primary THA or TKA at University Medical Center Groningen. Preoperative and one-year postoperative patients filled in the SQUASH questionnaire to get an impression of their physical activity level, and the WOMAC questionnaire to obtain insight into degree of recovery. Missing data were multiply imputed. No relationship was found between the preoperative total (B-coefficient = 0.03, CI95% = −0.033–0.093) and leisure-time physical activity level (B-coefficient = 0.042, CI95% = −0.009–0.093) neither for preoperative compliance with the Dutch Recommendation for Health-Enhancing Physical Activity (B-coefficient = 0.002, CI95% = −0.053–0.057), and the degree of recovery one year after surgery.

**Conclusion:**

The preoperative physical activity level had no relation with the degree of recovery one year after THA or TKA. The results do not support one of the major assumptions behind prevalidation, which assumes that a higher preoperative physical activity level will lead to a better recovery after THA or TKA.

## Introduction

Based on the Dutch GP registries, osteoarthritis (OA) is the most common progressive disorder of the musculoskeletal system [1], [2]. In the Netherlands, the prevalence of OA of the hip was 29.1 per 1,000 people and the prevalence of OA of the knee was 38.1 per 1,000 people [1], [2]. Total hip and total knee arthroplasty (THA and TKA, respectively) are both successful and cost-effective interventions in end-stage OA [3].

It is hypothesized that the amount of preoperative physical activity and thus the fitness level can have a positive effect on the postoperative recovery of patients undergoing THA or TKA. This assumption is based on the fact that physical activity, fitness and health are mutually influencing factors [4]. From these interrelationships it is hypothesized that preoperative exercise aimed at improving physical activity and fitness level – what is known as prevalidation – can be effective in improving postoperative recovery. Prevalidation is being introduced more and more in the treatment of THA and TKA patients with OA. Several Australian hospitals have already introduced preoperative physiotherapy programs for patients with OA who are on a waiting list for a joint replacement [5]. However, current literature is inconclusive about the effect of prevalidation. The most recent systematic review by Hoogeboom et al. [6] concluded that it remains unconfirmed whether prevalidation affects functional recovery after THA or TKA.

Objective of this study is to analyze one of the major assumptions underlying the potential effectiveness of prevalidation namely that the amount of preoperative physical activity and consequently the fitness level has a positive effect on the postoperative recovery of patients undergoing THA or TKA. Insight into this relationship can strengthen or weaken the arguments for using prevalidation as a tool to improve postoperative recovery. The main question in this research is: Is there a connection between preoperative physical activity level and degree of recovery one year after THA or TKA in patients with OA?

This research question is divided into three sub-questions:

1. Is there a connection between the preoperative total physical activity level and the degree of recovery one year after THA or TKA?
2. Is there a connection between the preoperative level of leisure-time physical activity and the degree of recovery one year after THA or TKA?
3. Is there a connection between preoperatively meeting the Dutch Recommendation for Health-Enhancing Physical Activity and the degree of recovery one year after THA or TKA?

## Method

### Design

A retrospective cohort study of prospectively collected data was conducted. Patients were asked to fill in the SQUASH and WOMAC questionnaires preoperatively and one year postoperatively. Completion of the questionnaire was taken as consent to participate. The Ethics Committee of University Medical Center Groningen approved this study (METc 2013/259).

### Participants

All patients that underwent a primary THA or TKA at University Medical Center Groningen between 2006 and 2012 were included. Patients who got a THA or TKA because of primary OA and had no operation within the following year after surgery were included in this study.

### Outcome measures

Level of physical activity and meeting the Dutch Recommendation for Health-Enhancing Physical Activity (NNGB) were measured with the SQUASH questionnaire [7]. The SQUASH is a self-reported questionnaire that measures habitual physical activity during a normal week over the past few months. The NNGB advises moderate-intensity physical activity for 30 minutes or more at least five days a week, which corresponds with the international standard as formulated by the American College of Sports Medicine and the American Heart Association [8]. The total score is reproduced as minutes per week. The SQUASH is considered reliable and valid in the general population [7] and in patients after THA [9].

Degree of recovery was measured with the WOMAC questionnaire. The WOMAC is a self-reported questionnaire that measures OA-caused constraints experienced by the patient in function and degree of pain and stiffness when performing daily activities. The total score is reproduced on a 100-point scale, where a higher score represents a lesser degree of perceived restriction in daily activities. The Dutch version of the WOMAC questionnaire is considered reliable and valid [10]. Demographic characteristics (age, gender, ASA classification, BMI, family status and education) were also assessed. For missing values, multiple imputation was used with the predictive mean matching model type, choosing to create 20 imputated datasets in this study so that the pooled results can be considered reliable [11], [12].

### Data analysis

Statistical analyses were performed with SPSS20. Descriptive statistics were used to describe the main characteristics. Univariate linear regression analysis was performed to answer the research questions. A second forward stepwise linear regression analysis was performed, adjusted for several potential confounders: type of surgery (THA or TKA), sex, BMI, ASA class and age of the patient at the time of surgery. A p-value of <0.05 was considered statistically significant.

## Results

### Flow of participants through the study

**Figure 1:**
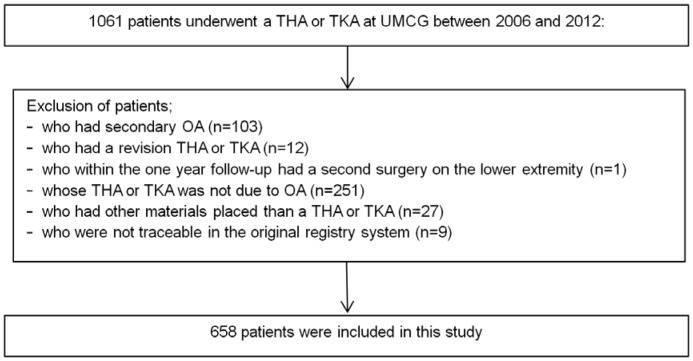
Flowchart of patient inclusion.

From the 1061 patients who had undergone a primary THA or TKA, 658 were included (see Fig. 1: flowchart).

### Baseline characteristics

The mean age was 68 years (SD 10.6), with more women than men (32%) and more THA than TKA (36%). The majority had finished a form of lower (22%) or secondary (50%) education (see Table 1). One year after THA or TKA, patients showed significant increases in minutes spent on leisure-time activity, and there was an 11% increase of patients who met the NNGB (see Table 2). The one-year recovery showed an increase of 37 points on the median WOMAC score (see Table 2). The distribution of the outcomes measures (level of physical activity in total and leisure time, and the degree of recovery) was skewed, therefore a log transformation was applied.

**Table 1:** Demographic characteristics.

	data
Sex (n. (%))	
male	211 (32)
female	447 (68)
Age (yrs) (mean ± SD*)	67.9±10.6
BMI (kg/m^2^) (mean ± SD*)	28.7±4.9
ASA class (n. (%))	
1	82 (12.4)
2	433 (65.8)
3	140 (21.2)
4	3 (0.4)
Highest level of education (n. (%))	
Lower **	146 (22)
Secondary ***	329 (50)
Higher ****	161 (25)
Other	22 (3)

*Standard deviation.

**Elementary school, vocational education.

***High school, intermediate vocational education.

****Higher professional education, university.

**Table 2:** Results of outcome measurements.

	Preoperatively	Postoperatively	Sig.
Total physical activity (minutes) (M(P25–P75))*	1140 (975–2040)	1525 (1200–1980)	0.118
Leisure-time physical activity (minutes) (M(P25–P75))*	270 (120–570)	420 (210–720)	<0.001
NNGB** compliance (n.(%))	189 (29)	262 (40)	0.005
Degree of recovery (M(P25–P75))*	41 (30–50)	78 (73–86)	<0.001

*M(P25–P75) = Median (25^th^ percentile −75^th^ percentile).

**NNGB = Dutch Recommendation for Health-Enhancing Physical Activity.

### Connection between preoperative physical activity level and degree of recovery

#### Preoperative total physical activity level and degree of recovery

There was no significant connection between the total preoperative physical activity level and the degree of recovery one year after surgery (B-coefficient = 0.03, CI95% = 0.033–0.093), even after adjustment for confounders (see Table 3).

**Table 3:** Results of forward stepwise linear regression in relation to degree of recovery, corrected for confounders*.

		B	SE	CI 0.25	CI 0.75
**Total preoperative level of physical activity**	Constant:	0.838	0.100	0.642	1.034
	Degree of recovery:	0.030	0.032	−0.033	0.093
**Leisure-time preoperative level of physical activity**	Constant:	0.832	0.064	0.707	0.957
	Degree of recovery;	0.042	0.026	−0.009	0.093
**Preoperative compliance with NNGB** **	Constant:	0.932	0.015	0.903	0.961
	Degree of recovery:	0.002	0.028	−0.053	0.057

*Confounders: surgical procedure (THA/TKA), age, gender, ASA classification, BMI, family status and educational level.

**NNGB = Dutch Recommendation for Health-Enhancing Physical Activity.

#### Preoperative leisure-time physical activity level and degree of recovery

There was no significant connection between the leisure-time preoperative physical activity level and the degree of recovery one year after surgery either (B-coefficient = 0.042, (CI95% = −0.009–0.093), even after adjustment for confounders (see Table 3).

#### Preoperatively meeting the NNGB for health-enhancing physical activity and degree of recovery

No significant connection was found between preoperatively meeting the NNGB and the degree of recovery one year after surgery (B-coefficient = 0.002, CI95% = –0.053–0.057), even after adjustment for confounders (see table 3).

## Discussion

The basis for this study is the lack of understanding of the connection between preoperative physical activity level and degree of recovery after THA or TKA. Available studies report conflicting results [5], [6], [13]. Therefore the objective of this study was to analyze one of the major assumptions underlying the potential effectiveness of prevalidation namely the fact that the amount of preoperative physical activity can have a positive effect on the postoperative recovery of patients undergoing THA or TKA. Based on the 658 patients in our study, all of whom underwent THA or TKA, we concluded that the preoperative physical activity level is not related to the degree of functional recovery one year postoperatively. This applies to the total physical activity level as well as to the level of physical activity during leisure time and to whether or not the patient meets NNGB norms preoperatively, even after correcting for confounders such as surgical procedure (THA or TKA), sex, BMI, ASA class and age of the patient. In that sense it can be concluded that the results do not support the major assumption underlying the potential effectiveness of prevalidation assuming that a higher preoperative physical activity level leads to a positive effect on the extent of recovery after THA or TKA. The fact that, among the confounders, neither ASA class nor age are of influence is remarkable, as in other studies it is argued that prevalidation is especially effective in older and more vulnerable patients [14].

The results of this study are in accordance with recent reviews on the link between prevalidation and degree of recovery after THA or TKA [5], [6], [13] in which no effect could be found. Dauty et al. [13] concluded that prevalidation leads to a higher preoperative degree of physical functioning, and, as in our study, a higher preoperative degree of physical functioning did not lead to a higher degree of recovery, where recovery is defined as the degree of restriction experienced in normal daily activities. Hoogeboom et al. [6] indicate as a possible explanation that prevalidation programs in most studies do not meet the requirements for therapeutic validity. The studies do not describe in advance what an optimal program would be. In only three studies were patients offered a program with a higher physical activity level than the weekly amount considered according to the standard physical activity norm. Hoogeboom et al. [6] also state that there was no monitoring of the intensity of the program. On the other hand it can also be argued that in our study patients did not participate in a specific prevalidation program at all, but that we only looked into the amount of physical activity. However the outcome of our results gathered with validated questionnaires does not show a connection between preoperative physical activity level and postoperative recovery, hence it doesn’t support the assumption behind prevalidation.

A strength of this study is the relatively large group of 658 patients who had undergone THA or TKA. Because all THA and TKA patients in the registries of UMCG are included, no selection bias occurred. In many studies on the effect of prevalidation a relatively large number of patients drop out during the study. Rooks et al. [15] concluded that patients who drop out during a study are not representative of the research: the dropouts were older, had a lower BMI, reported more pain and had a lower functional capacity. In the course of our study no patients dropped out. Patients whose data was yet incomplete at the time of the analysis were not removed from the study, but valid inferences were made by multiple imputation [12]. On the other hand, it should be noted that the patient population comes from an orthopaedic department from one single university medical centre, therefore the results should not be directly generalized to the entire Dutch population. In addition it should be remarked that because of the large group of patients a self-reported questionnaire was used to measure physical activity. From literature it is known that compared to objective measures of physical activity, overestimation is an intrinsic property of self-assessment questionnaires like the SQUASH. For that it is not advised to use questionnaires on a individual level, however these can be used on a group level as was done in this study [16], [17].

Overall it can be concluded that in this study preoperative physical activity level is not related to the degree of recovery one year after THA or TKA in patients with primary OA. It must however be taken into consideration that in this study the level of recovery was measured at one single moment, namely one year postoperatively. Patients are generally considered to have completely recovered one year after THA or TKA, yet this offers no insight into the connection between preoperative physical activity level and speed of recovery. Whitney et al. [18] stated that patients with a higher preoperative physical activity level have a higher physical activity level in the first days after surgery, which is why they are discharged earlier from the hospital and go faster through their rehabilitation process. For future research it would be interesting to focus on the speed of recovery, the more so because increasing numbers of patients are undergoing THA or TKA at a younger age. These patients often play an active role in society and in the labour market, so for their sake and that of society it is of great importance to recover quickly. If prevalidation is found to be effective, leading to a faster recovery and thus a faster return to the active community, it will be a great opportunity for physiotherapists, orthopaedic surgeons and health insurers. We therefore recommend a follow-up study, not only to examine the link between preoperative physical activity level and amount of recovery in the early stages, but most importantly to assess the speed of recovery by taking multiple measuring points along the recovery process.

## Supporting Information

S1 Data Multiple imputed dataset.
(SAV)
